# Identification of the first small-molecule ligand of the neuronal receptor sortilin and structure determination of the receptor–ligand complex

**DOI:** 10.1107/S1399004713030149

**Published:** 2014-01-29

**Authors:** Jacob Lauwring Andersen, Tenna Juul Schrøder, Søren Christensen, Dorthe Strandbygård, Lone Tjener Pallesen, Maria Marta García-Alai, Samsa Lindberg, Morten Langgård, Jørgen Calí Eskildsen, Laurent David, Lena Tagmose, Klaus Baek Simonsen, Philip James Maltas, Lars Christian Biilmann Rønn, Inge E. M. de Jong, Ibrahim John Malik, Jan Egebjerg, Jens-Jacob Karlsson, Srinivas Uppalanchi, Durga Rao Sakumudi, Pradheep Eradi, Steven P. Watson, Søren Thirup

**Affiliations:** a The Lundbeck Foundation Research Centre MIND, Department of Molecular Biology and Genetics, Aarhus University, Gustav Wieds Vej 10C, 8000 Aarhus C, Denmark; b Neuroscience Drug Discovery, H. Lundbeck A/S, Ottiliavej 9, 2500 Valby, Denmark; c The Lundbeck Foundation Research Centre MIND, Department of Biomedicine, Aarhus University, Ole Worms Allé 3, 8000 Aarhus C, Denmark; d Medicinal Chemistry, GVK BioScience, Plot No. 28 A, IDA Nacharam, Hyderabad 500 076, India

**Keywords:** sortilin, small molecules, ligands, AF40431, proNGF, Alzheimer’s disease, Vps10p

## Abstract

Sortilin is a type I membrane glycoprotein belonging to the vacuolar protein sorting 10 protein (Vps10p) family of sorting receptors and is most abundantly expressed in the central nervous system. Sortilin has emerged as a key player in the regulation of neuronal viability and has been implicated as a possible therapeutic target in a range of disorders. Here, the identification of AF40431, the first reported small-molecule ligand of sortilin, is reported. Crystals of the sortilin–AF40431 complex were obtained by co-crystallization and the structure of the complex was solved to 2.7 Å resolution. AF40431 is bound in the neurotensin-binding site of sortilin, with the leucine moiety of AF40431 mimicking the binding mode of the C-terminal leucine of neurotensin and the 4-methyl­umbelliferone moiety of AF40431 forming π-stacking with a phenylalanine.

## Abstract 2

The identification of the first small-molecule ligand of the neuronal receptor sortilin and structure determination of the receptor–ligand complex are reported.

## 1. Introduction   

Sortilin is a 100 kDa type I membrane glycoprotein belonging to the vacuolar protein sorting 10 protein (Vps10p) receptor family. The receptor is composed of a large extracellular domain (75 kDa), a single transmembrane helix and a short cytoplasmic tail (Petersen *et al.*, 1997 ▶). The extracellular Vps10p domain is composed of a β-propeller and a cysteine-rich (10CC) domain (Quistgaard *et al.*, 2009 ▶). The β-propeller is composed of ten repeats (blades), forming a wheel-like structure with a funnel-shaped tunnel with a diameter of 25–­40 Å (Fig. 4). Each blade is composed of a four-stranded β-­sheet and is stabilized by an Asp-box repeat in the loop between strands 3 and 4 (Quistgaard & Thirup, 2009 ▶). Sortilin is a key player in the regulation of neuronal viability and function and is widely expressed in the central nervous system (CNS) during development and in adulthood (Petersen *et al.*, 1997 ▶; Hermans-Borgmeyer *et al.*, 1999 ▶). The receptor is mainly located in intracellular membrane structures (endoplasmic reticulum, trans-Golgi network and endosomal organelles) involved in the sorting of ligands. It has been estimated that a tenth of the cellular sortilin pool is found at the cell surface (Mazella *et al.*, 1998 ▶), mediating endocytosis of ligands through clathrin-coated pits (Nielsen *et al.*, 2001 ▶). The 13-­amino-acid neuropeptide neurotensin (NTS) is a ligand of the G-coupled neurotensin receptors (NTSRs), resulting in dopamine turnover and muscle relaxation, but is also bound by sortilin in the extracellular environment (Munck Petersen *et al.*, 1999 ▶). NTS is bound to sortilin in the centre of the tunnel of the β-propeller and the binding site is composed of a β-­strand interaction between the C-terminus (amino acids 10–13) of neurotensin and strand 1 of blade 6 in sortilin. A strong salt bridge is formed between the carboxylic acid of the C-­terminus and Arg292 (Quistgaard *et al.*, 2009 ▶).

A notable function of sortilin is its role in signalling by neurotrophins, a family consisting of nerve growth factor (NGF), brain-derived neurotrophic factor (BDNF) and neurotrophins 3 and 4 (S-3 and NT-4) in humans. The hallmark of neurotrophin signalling, neuronal survival, is mediated through binding of mature neurotrophin to tyrosine receptor kinases (Trks; Wu *et al.*, 2002 ▶). The affinity and specificity of neurotrophin binding is modulated by the presence of the low-affinity neurotrophin receptor p75 (p75NTR) acting as a co-receptor (Esposito *et al.*, 2001 ▶; Hempstead *et al.*, 1991 ▶; Bibel *et al.*, 1999 ▶). Neurotrophins are expressed with a pro-domain, which is cleaved off in the Golgi, resulting in secretion of the mature domain (Scott *et al.*, 1983 ▶). However, the release of unprocessed proneurotrophins results in signalling of apoptosis *via* p75NTR (Lee *et al.*, 2001 ▶) and sortilin (Nykjaer *et al.*, 2004 ▶; Teng *et al.*, 2005 ▶; Tauris *et al.*, 2011 ▶). The mature part of the neurotrophin is bound by p75NTR, whereas the propeptide is bound by sortilin, thereby forming a high-affinity (*K*_d_ of ∼160 p*M*) heterotrimeric complex (Nykjaer *et al.*, 2004 ▶). Knockout studies have supported the roles of p75NTR (Frade & Barde, 1999 ▶) and sortilin (Jansen *et al.*, 2007 ▶) in proneurotrophin-induced apoptosis signalling. Proneurotrophins are released to the extracellular environment in animal models of neurodegenerative conditions, including mechanical damage to the nervous system, focal ischaemia, axotomy, stroke and epileptic seizures (Dechant & Barde, 2002 ▶), and proNGF is detected *in vivo* for several days following injury (Jansen *et al.*, 2007 ▶). Elevated levels of proNGF (Harrington *et al.*, 2004 ▶), p75NTR (Beattie *et al.*, 2002 ▶) and sortilin (Jansen *et al.*, 2007 ▶) are observed in acute injury models. These upregulations have been suggested to keep the nervous system functional by the clearance of damaged neurons (Lu *et al.*, 2005 ▶). Interestingly, elevated levels of proNGF have been identified in patients diagnosed with Alzheimer’s disease (Fahnestock *et al.*, 2001 ▶). The levels of proneurotrophin in the extracellular environment are controlled by plasmin and matrix metalloproteases (Lee *et al.*, 2001 ▶). Elevated levels of inhibitors of the extracellular proteases are found under conditions resembling neuro­degenerative diseases (Bruno & Cuello, 2006 ▶).

Outside the CNS, proNGF is overproduced and secreted from malignant breast cancer cells, thereby inducing autocrine stimulation *via* TrkA and sortilin (Demont *et al.*, 2012 ▶), and similar autocrine stimulation has been observed in melanoma cells (Truzzi *et al.*, 2008 ▶). Sortilin has further been demonstrated to interact with apolipoprotein B100 in the Golgi and to facilitate the export of apoB100-containing lipoproteins, thereby regulating the levels of low-density lipoprotein (LDL) cholesterol, a key contributor to atherosclerosis and ischaemic heart disease, in plasma (Kjolby *et al.*, 2010 ▶).

The interaction between sortilin and the majority of its ligands, including the proneurotrophins, is inhibited by NTS (Nykjaer *et al.*, 2004 ▶; Munck Petersen *et al.*, 1999 ▶). Hence, a small-molecule ligand mimicking the binding mode of NTS could potentially be applied as a treatment in several disorders in which sortilin has been implicated. Here, we describe the identification of the first small-molecule ligand of sortilin, AF40431, and the structure determination of the receptor–ligand complex.

## 2. Materials and methods   

### 2.1. Synthesis and characterization of AF40431 and **1h**   

Compounds **1a**–**1g** were obtained directly from the H. Lundbeck compound collection. AF40431 and **1h** were prepared by reductive alkylation and deprotection of l- and d-­leucine *tert*-butyl esters with the corresponding coumarin aldehyde (7-hydroxy-4-methyl-2-oxo-2*H*-chromene-8-carb­aldehyde).

Sodium triacetoxyborohydride [NaBH(OAc)_3_] (1.55 g, 7.34 mmol) was added to a solution of l-leucine *tert*-butyl ester hydrochloride (0.50 g, 2.44 mmol) and the coumarin aldehyde (0.68 g, 2.93 mmol) in dichloroethene (DCE; 50 ml) and acetic acid (0.14 g, 2.44 mmol) and the reaction mixture was stirred at room temperature for 16 h. Aqueous sodium bicarbonate (NaHCO_3_) solution (15 ml) was added and the mixture was extracted with ethyl acetate (2 × 50 ml), washed with brine and dried over anhydrous sodium sulfate (Na_2_SO_4_). Solvents were removed *in vacuo* and the crude compound was purified by silica gel (100–200 mesh) column chromatography, eluted with 16% of ethyl acetate in petroleum ether, to afford the *tert*-butyl ester of AF40431 (0.37 g, 40%) as a pale yellow solid. ^1^H NMR (CDCl_3_, 600 MHz, TMS) δ: 7.43–7.41 (1H, d, *J* = 8.7 Hz), 6.80–6.78 (1H, d, *J* = 8.7 Hz), 6.07 (1H, s), 4.30–4.26 (1H, d, *J* = 14.9 Hz), 4.18–4.15 (1H, d, *J* = 14.9 Hz), 3.25–3.23 (1H, t, *J* = 7.1 Hz), 2.38 (3H, br s), 1.78–1.71 (1H, sp, *J* = 6.1 Hz), 1.56–1.49 (2H, m), 1.51 (9H, s), 0.95–0.93 (3H, d, *J* = 6.6 Hz), 0.92–0.90 (3H, d, *J* = 6.6 Hz).

The *tert*-butyl ester of AF40431 (0.150 g, 0.39 mmol) was taken up in 1:1 dichloromethane (DCM)/trifluoroacetic acid (TFA) (2 ml) and stirred at room temperature for 3 h. The solvents were removed *in vacuo* and the crude compound was washed with diethyl ether (2 × 5 ml) and methanol (2 × 5 ml) and dried *in vacuo* to afford AF40431 (0.091 g, 72%) as a white solid. ^1^H NMR (DMSO-d_6_, 600 MHz, TMS) δ: 9.95–8.95 (1H, br s), 7.58–7.56 (1H, d, *J* = 8.8 Hz), 6.82–6.80 (1H, d, *J* = 8.8 Hz), 6.15 (1H, s), 4.14–4.10 (1H, d, *J* = 14.4 Hz), 4.04–4.00 (1H, d, *J* = 14.4 Hz), 3.27–3.22 (1H, m), 2.37 (3H, s), 1.78–1.71 (1H, sp, *J* = 6.5 Hz), 1.53–1.43 (2H, m), 0.89–0.87 (3H, d, *J* = 6.5 Hz), 0.86–0.84 (3H, d, *J* = 6.5 Hz). Mass spectrum: tR = 0.50 min, *m*/*z* = 320.0 (*M* + H)^+^.

Compound **1h** was prepared in an analogous manner to AF40431, starting from d-leucine *tert*-butyl ester hydrochloride. ^1^H NMR spectra were recorded at 600 MHz using a Bruker Avance AV-III-600 instrument. Chemical shift values are expressed in p.p.m. relative to tetramethylsilane. The following abbreviations or their combinations are used for the multiplicity of NMR signals: br, broad; d, doublet; m, multiplet; obs, obscured; s, singlet, sp, septet; t, triplet. LC-MS was run on a Waters Aquity UPLC-MS consisting of a column manager, a binary solvent manager, a sample organizer, a PDA detector (operating at 254 nm), an ELS detector and an SQ-MS equipped with an APPI source operating in positive-ion mode. The column was an Acquity UPLC BEH C18 (1.7 µm, 2.1 × 50 mm) operating at 60°C with a 1.2 ml min^−1^ binary gradient consisting of water/0.1% formic acid (solvent *A*) and acetonitrile/5% water/0.1% formic acid (solvent *B*). The gradient changes between 10% solvent *B* and 99.9% solvent *B* over the 1 min run.

### 2.2. Chiral supercritical fluid chromatography   

Chiral supercritical fluid chromatography (SFC) was run on an Aurora Fusion A5 unit together with Agilent 1100/1260 modules using a Phenomenex Lux 3u Cellulose-2 column, 4.6 × 250 mm, flow 3 ml min^−1^ under 15 MPa pressure at 40°C. Ethanol and 0.1% diethylamine were used as modifiers at 20% concentration and UV detection operated at 254 and 230 nm. In order to demonstrate that the compounds were enantiomerically pure, AF40431 and **1h** were separately treated with TMS-CH_2_N_2_ in THF to afford the corresponding methyl esters with identical NMR spectra: ^1^H NMR (CDCl_3_, 600 MHz, TMS) δ: 7.43–7.41 (1H, d, *J* = 8.5 Hz), 6.81–6.79 (1H, d, *J* = 8.5 Hz), 6.08 (1H, d, *J* = 1.1 Hz), 4.37–4.34 (1H, d, *J* = 14.1 Hz), 4.11–4.07 (1H, d, *J* = 14.1 Hz), 3.87 (3H, s), 3.42–3.38 (1H, m), 2.39–2.38 (3H, d, *J* = 1.2 Hz), 1.79–1.71 (1H, sp, *J* = 6.8 Hz), 1.58–1.55 (2H, obs m), 0.95–0.94 (3H, d, *J* = 6.8 Hz), 0.93–0.92 (3H, d, *J* = 6.8 Hz). Mass spectrum: tR = 0.45 min, *m*/*z* = 334.1 (*M* + H)^+^. These methyl esters were submitted to chiral SFC both in their pure forms and as a 1:1 mixture of enantiomers. The pure sample of the methyl ester derivatives of AF40431 and **1h** displayed unique retention times (tR = 2.85 and 3.34 min, respectively) by chiral SFC, while two distinct signals were observed for the mixed sample (tR = 2.84 and 3.33 min). As the homochiral starting materials l- and d-leucine afforded the homochiral products AF40431 and **1h**, respectively, it can be inferred that AF40431 has the (*S*) absolute configuration of l-leucine; as to not do so would have required complete stereochemical inversion during one of the chemical transformations, which would be both highly unlikely and unprecedented for these transformations. The absolute configuration of AF40431 was not unambiguously characterized.

### 2.3. Sortilin expression and purification   

The open reading frame of human sortilin including the endogenous signal peptide to residue 723 was amplified by PCR and cloned into the pCEP-pu vector with *Xba*I and *Xho*I. The primers were designed to include a C-terminal factor Xa site followed by a hexahistidine tag. HEK293F cells were transfected with 900 µg plasmid, applying 900 µl FreeStyle MAX reagent (Invitrogen) according to the manufacturer’s instructions, and were grown in 900 ml FreeStyle 293 medium (Invitrogen) in a 3 l Erlenmeyer flask for 7 d. The medium was harvested by centrifugation at 3700*g* for 15 min, filtered through a 0.40 µm filter and the pH was adjusted by supplementation with Tris–HCl pH 8.0 to a final concentration of 50 m*M*. A 5 ml Ni–NTA column (GE Healthcare) was equilibrated in buffer *A* (50 m*M* Tris–HCl pH 8.0, 150 m*M* NaCl) and the medium was recirculated over the column overnight. The column was washed with five column volumes of wash buffer (50 m*M* Tris–HCl pH 8.0, 150 m*M* NaCl, 10 m*M* imidazole) and sortilin was eluted with a ten-column-volume imidazole gradient (10–250 m*M* imidazole). Fractions containing sortilin were pooled and concentrated by ultracentrifugation (Vivaspin 20, 10 kDa cutoff, Sartorius). The sortilin was applied onto a Superdex 200 10/300 GL column (GE Healthcare) equilibrated in buffer *A*. Fractions containing sortilin were pooled, concentrated to a final concentration of 5 mg ml^−1^, flash-frozen in liquid nitrogen and stored at −80°C.

### 2.4. Scintillation proximity assay   

The compound affinity was determined by measuring the displacement of ^3^H-neurotensin binding to sortilin using a scintillation proximity assay (SPA). Experiments were performed in assay buffer (50 m*M* HEPES pH 7.4, 100 m*M* NaCl, 2.0 m*M* CaCl_2_, 0.1% BSA, 0.1% Tween-20) with a total volume of 40 µl. The compound was pre-incubated for 30 min at room temperature with 150 n*M* sortilin before 5 n*M* ^3^H-­neurotensin and Ni-chelate imaging beads (Perkin Elmer) were added. After 6 h, the plate was read on a ViewLux with 360 s exposure time. Unlabelled neurotensin and a DMSO blank were used as positive and negative controls, respectively. Dose-response evaluation of compounds was performed with ten concentrations of **1a**–**1h** and AF40431 (covering concentrations between 2.5 n*M* and 50 µ*M*). The half-maximal inhibitory concentration (IC_50_) values were calculated by nonlinear regression using the sigmoid concentration response (variable slope) in *XLfit* 4 (IDBS, UK). All values reported are the averages of at least four determinations.

### 2.5. Isothermal titration calorimetry   

The binding of AF40431 to sortilin was measured by isothermal titration calorimetry (ITC). The titration experiments were performed on a MicroCal iTC200 isothermal titration calorimeter (MicroCal, Northampton, Massachusetts, USA). Sortilin was dialysed against PBS buffer pH 7.4. AF40431 was dissolved in PBS buffer pH 7.4 supplemented with 4%(*v*/*v*) dimethyl sulfoxide (DMSO). All solutions were filtered and degassed to avoid bubble formation and were equilibrated to the corresponding temperature before each experiment. Sortilin (30 µ*M*) supplemented with 4%(*v*/*v*) DMSO was titrated at 25°C with AF40431 (500 µ*M*) in 20 steps of 2 µl (first step, 0.4 µl). The time between injections was set to 150 s and the syringe mixing speed was set to 1000 rev min^−1^. Heat evolving from dilution was measured by injecting the ligand into PBS supplemented with 4% DMSO. This heat was subtracted from the heat of reaction to obtain the effective heat of binding. Finally, the binding stoichiometry (*n*), equilibrium dissociation constant (*K*_d_), molar enthalpy and entropy changes for the binding processes were determined by analyzing the titration data using *Origin* software (OriginLab, Northampton, Massachusetts, USA).

### 2.6. AF40431 fluorescence spectroscopy   

AF40431 and sortilin were diluted in PBS buffer pH 7.4 to a final concentration of 0.1 µ*M*. Fluorescence was recorded on a RF-5301PC spectrofluorophotometer (Shimadzu) maintained at 23 ± 1°C using 3 and 5 nm excitation and emission slit widths, respectively. The excitation and emission wavelengths were determined by scanning the ranges 250–430 and 370–600 nm, respectively.

### 2.7. Sortilin crystallization, data collection and refinement   

Initial screening was performed using in-house polyethylene glycol (PEG) screens designed with the *Mimer* spreadsheet (Brodersen *et al.*, 2013 ▶). Sortilin was pre-incubated with a ten-molar excess of AF40431 for 1 h. 200 nl sortilin–AF40431 was mixed with 200 nl reservoir solution using a Mosquito liquid-handling robot (TTP LabTech) and equilibrated against 100 µl reservoir solution using the sitting-drop vapour-diffusion method at 292 K. Several crystal hits were obtained and were further optimized by grid screening and varying the molar excess of AF40431 in 24-well crystallization plates. The best diffracting crystals were obtained with a 20-molar excess of AF40431 in 0.1 *M* HEPES–Tris pH 7.3, 0.4 *M* sodium malonate, 26%(*w*/*v*) PEG 3350 (Hampton Research), 8%(*v*/*v*) glycerol. The crystals grew to dimensions of 200 × 100 × 50 µm over two weeks. The crystals were mounted in LithoLoops (Molecular Dimensions) from the mother liquor, excess mother liquor was removed by gently touching the side of the well with the edge of the loop (Morth *et al.*, 2007 ▶) and the crystals were flash-cooled in liquid N_2_. A complete single-wavelength (λ = 0.9 Å) data set of 1100 oscillation images with 0.2° oscillation was collected at 100 K on the X06DA (PXIII) beamline at the Swiss Light Source (SLS) using a PILATUS 2M-F detector (Dectris). The diffraction images were processed in *XDS* (Kabsch, 2010 ▶) and the reflections were scaled in *SCALA* (Winn *et al.*, 2011 ▶). Molecular replacement was performed with *Phaser* (McCoy *et al.*, 2007 ▶) using a search model derived from the structure of sortilin in complex with neurotensin (PDB entry 3f6k; Quistgaard *et al.*, 2009 ▶). Rigid-body refinement, generation of ligand coordinates and restraints, calculation of OMIT maps and refinement were performed in *PHENIX* (Adams *et al.*, 2010 ▶). Model building and analysis was performed using *Coot* (Emsley *et al.*, 2010 ▶). The final model quality was analysed using *MolProbity* (score of 11.77; Chen *et al.*, 2010 ▶). R.m.s.d. values were calculated, superpositions were performed and structural figures were prepared using *PyMOL* (Schrödinger; http://www.pymol.org).

## 3. Results   

### 3.1. Identification of AF40431   

**Figure 1:**
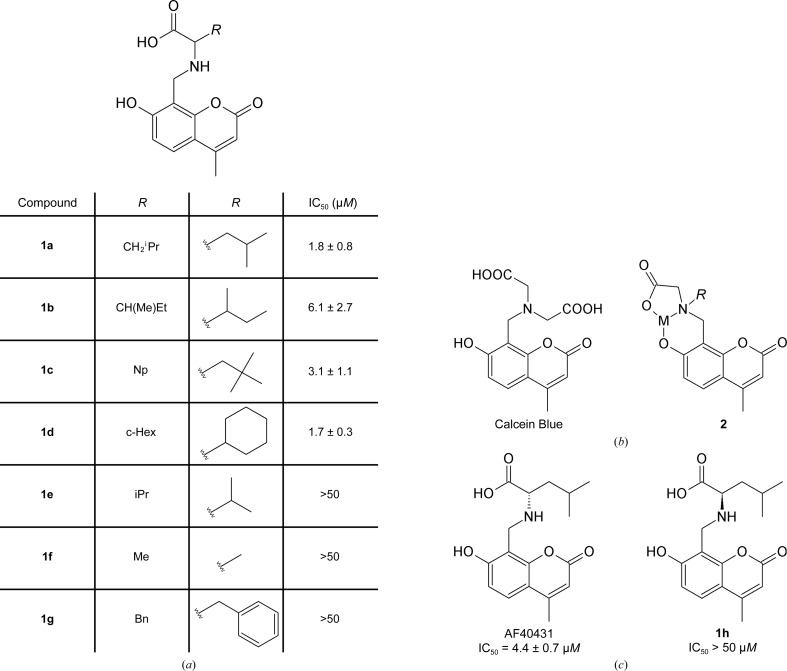
Identification of AF40431. (*a*) Chemical structure and half-maximal inhibitory concentration (IC_50_) values determined in the SPA assays of the hit compound **1a** and its analogues (**1b**–**1g**). The stereochemistry of the samples in the compound collection was undefined [*i.e.* (+), (−) or (±)]. (*b*) Chemical structure of Calcein Blue (left) and tridentate metal (M) chelation by Calcein Blue (right). (*c*) Chemical structure and IC_50_ values determined from the SPA assay of AF40431 and its stereoisomer **1h**. Chemical structure were drawn using *ChemSketch* (ACD/Labs; http://www.acdlabs.com).

**Figure 2:**
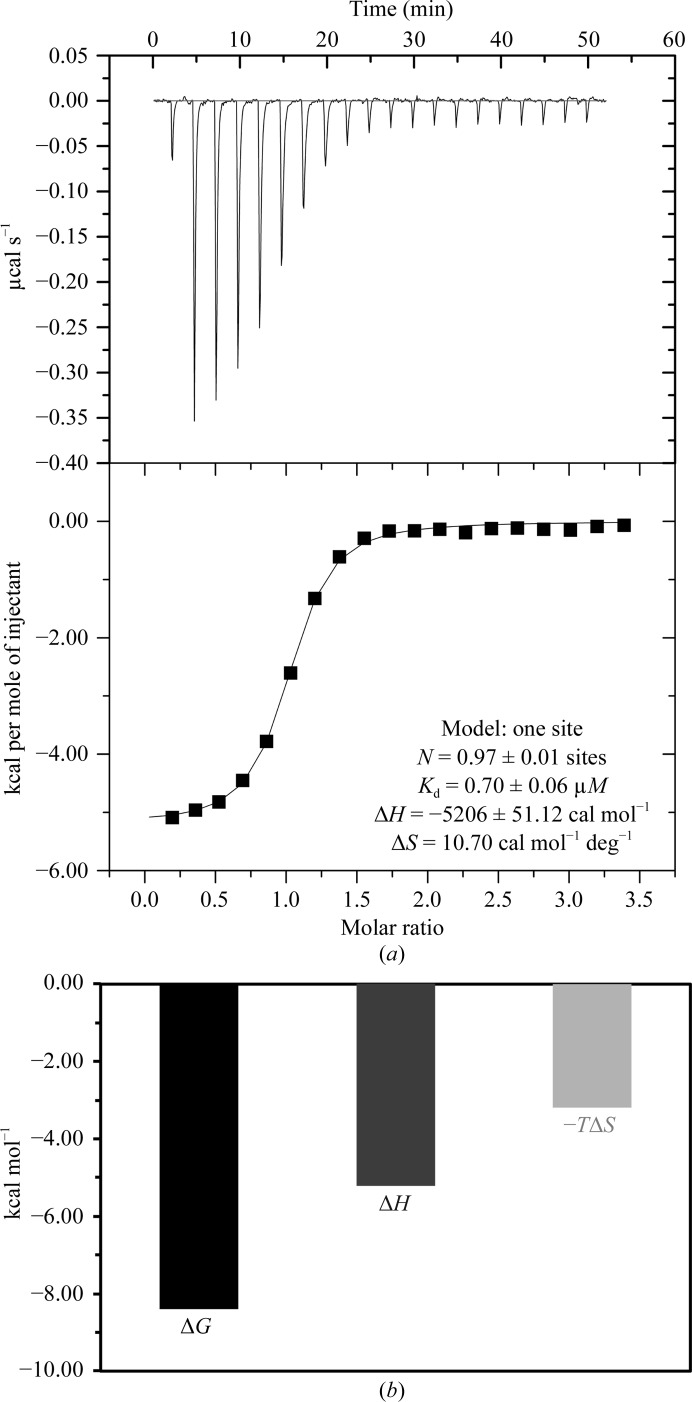
Thermodynamics of the binding of AF40431 to sortilin. (*a*) The raw titration data and the integrated titration curves are depicted in the upper and lower panels, respectively. The final values of the determined thermodynamic parameters are listed in the lower panel. (*b*) Binding signature plot listing the enthalpic (Δ*H*) and entropic (−*T*Δ*S*) contributions to the free binding energy (Δ*G*).

Compound **1a**, a promising hit with an IC_50_ of 1.8 µ*M* (Fig. 1 ▶ *a*), was identified in a high-throughput screen of the H. Lundbeck compound collection using the ^3^H-neurotensin-binding sortilin scintillation proximity assay (SPA). **1a** is closely related to the dye Calcein Blue (Fig. 1 ▶ *b*) and could thus potentially cause assay interference by fluorescence. Furthermore, molecules of the general type **1** have been reported (Wilkins, 1960 ▶) to ligate metals *via* a tridentate binding interaction (compound **2**; Fig. 1 ▶ *b*), which could also potentially cause assay interference. However, screening of analogues of **1a** revealed a promising structure–activity relationship, which was consistent with specific molecular recognition rather than chemical-mediated assay interference (Fig. 1 ▶ *a*). Specifically, the activity was retained when the leucine-type side chain of **1a** (CH_2_^i^Pr) was exchanged to the isoleucine-type side chain of **1b** [CH(Me)Et], the neopentyl substituent of **1c** or the cyclohexyl substituent of **1d**. In contrast, the activity was ablated by either substitution with the more compact valine- and alanine-type side chains of **1e** (^i^Pr) and **1f** (Me), respectively, or the bulkier phenylalanine-type side chain of **1g** (Bn). The stereochemistry of the samples in the compound collection (**1a**–**1g**) was undefined and therefore the influence of the stereochemistry on the activity was explored for **1a**. The activity was found to reside in only one enantiomer, AF40431, whereas the corresponding antipode **1h** was inactive (Fig. 1 ▶ *c*). Such enantiospecific activity is consistent with a genuine molecular-recognition event with the target protein rather than assay interference, which would be expected to afford similar effects for both enantiomers. The activity of racemic sample **1a** and the homochiral AF40431 were identical within experimental error. The binding of AF40431 to sortilin was furthermore confirmed by ITC with a dissociation constant (*K*_d_) of 0.7 µ*M* (Fig. 2 ▶ *a*), and the free binding energy is driven by both enthalpic and entropic contributions (Fig. 2 ▶ *b*). These structure–activity relationships gave us sufficient confidence in AF40431 to attempt co-crystallization with sortilin.

### 3.2. Crystallization of the sortilin–AF40431 complex   

The extracellular Vps10p domain of sortilin was overexpressed in HEK293F cells and purified from the medium by immobilized metal-affinity chromatography. Sortilin was further purified by size-exclusion chromatography, eluting as a single symmetrical peak corresponding to monomeric sortilin. Crystals of the sortilin–AF40431 complex were obtained by co-crystallization. Initial crystallization hits were identified in an in-house polyethylene glycol (PEG) screen and were further optimized by grid screening and increasing the molar excess of AF40431. A complete data set was collected, scaling to a maximum resolution of 2.7 Å. The crystals of the sortilin–AF40431 complex belonged to the monoclinic space group *C*2, with unit-cell parameters *a* = 160.9, *b* = 79.2, *c* = 111.7 Å, β = 127.3°. The data statistics are summarized in Table 1 ▶.

### 3.3. Structure determination of the sortilin–AF40431 complex   

**Figure 3:**
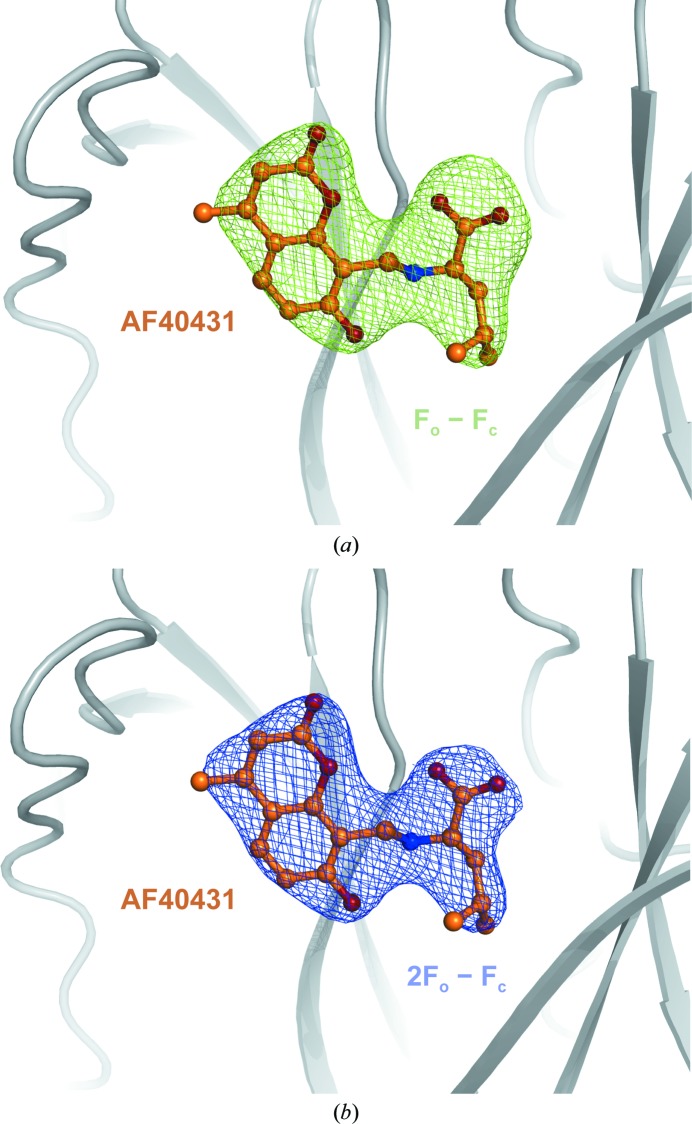
Electron-density maps of AF40431. (*a*) Bias-reduced simulated-annealing *F*_o_ − *F*_c_ map contoured at the 3.0σ level. (*b*) Final 2*F*_o_ − *F*_c_ map contoured at the 1.5σ level.

The atomic coordinates of the published sortilin–neurotensin complex structure (PDB entry 3f6k; Quistgaard *et al.*, 2009 ▶), stripped of NTS, glycosylations and waters, was applied as a search model to obtain initial phases by molecular replacement (MR), yielding a translation-function *Z*-score (TFZ) of 66.5. The asymmetric unit was composed of a single molecule. The molecular-replacement solution was subjected to rigid-body and simulated-annealing refinement. Previously identified glycosylations (Quistgaard *et al.*, 2009 ▶) were easily identified in the resulting bias-reduced *F*_o_ − *F*_c_ electron-density maps, thereby validating the MR solution. Furthermore, additional density was observed in the NTS-binding site in the bias-reduced *F*_o_ − *F*_c_ electron-density maps which remained during the building of glycosylations and subsequent rounds of refinement. Coordinates and geometric restraints were generated for AF40431, and the ligand could unambiguously be modelled into the density observed at the NTS-binding site. The structure was refined, resulting in good *R* factors (*R*_work_ and *R*_free_ of 20.7% and 22.8%, respectively; Table 1 ▶). Binding of AF40431 was confirmed by bias-reduced simulated-annealing OMIT maps (Fig. 3 ▶ *a*) and the density for AF40431 was well defined in the final 2*F*_o_ − *F*_c_ maps (Fig. 3 ▶ *b*).

### 3.4. AF40431 binding to sortilin   

**Figure 4:**
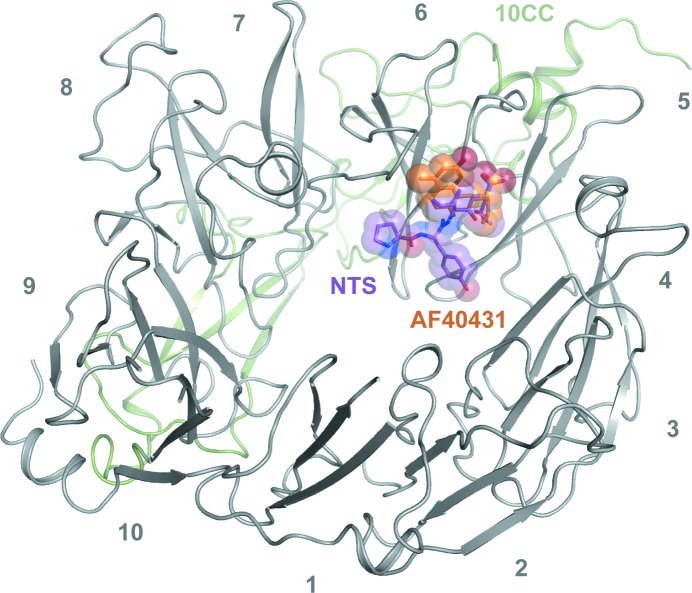
Overview of AF40431 binding to sortilin. The ten blades of the β-­propeller and the 10CC domain are represented as grey and green cartoons, respectively. Neurotensin was superimposed from PDB entry 3f6k (Quistgaard *et al.*, 2009 ▶) and is depicted as a purple ball-and-stick representation; AF40431 is depicted as orange sticks and spheres.

**Figure 5:**
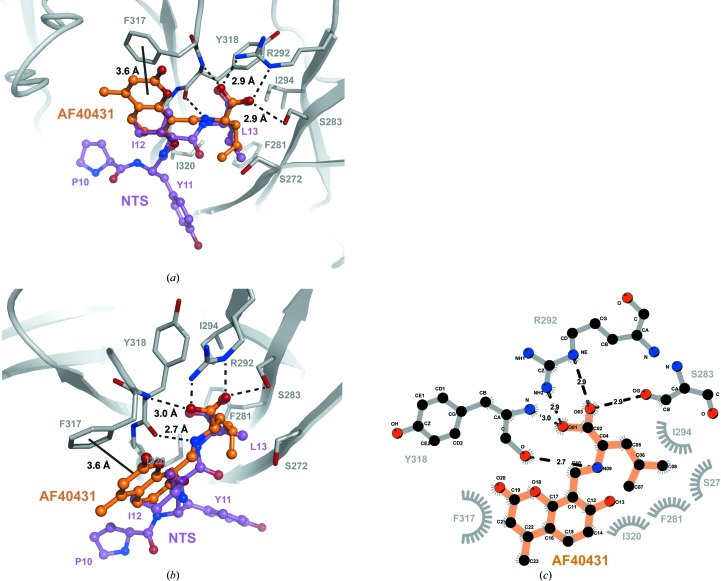
Binding mode of AF40431 to sortilin. (*a*, *b*) Sortilin is represented as a cartoon in grey and the residues interacting with AF40431 are represented as sticks. C atoms of sortilin, AF40431 and NTS are depicted in grey, orange and purple, respectively, and N and O atoms are depicted in blue and red, respectively. (*c*) Schematic diagram of hydrogen bonds (black punctured lines) and hydrophobic interactions (grey arches with spokes) between AF40431 and sortilin prepared in *LigPlot* ^+^ (Laskowski & Swindells, 2011 ▶).

AF40431 is bound to sortilin in the centre of the tunnel of the β-propeller at the NTS-binding site (Fig. 4 ▶). The overall structure of the AF40431–sortilin complex resembled that of the NTS–sortilin complex (r.m.s.d. of 0.55 Å for C^α^ atoms), with the 10CC domain wrapped around the β-propeller. The leucine moiety of AF40431 is bound to sortilin in the same way as that of NTS, with the carboxylic acid forming a salt bridge to Arg292. The orientation of the carboxylic acid is further stabilized by hydrogen bonds to Ser283 and to the amide of Tyr318 (Fig. 5 ▶ *a*). A hydrogen bond is formed between the secondary amine of AF40431 and the carbonyl of Tyr318 (Fig. 5 ▶ *b*). The isopropyl group of AF40431 is buried in the hydrophobic pocket formed by Ser272, Phe281, Ile294 and Ile320, but is rotated 120° compared with that of NTS. The 2-­pyrone of the 4-methylumbelliferone moiety of AF40431 forms an off-centre parallel π-stacking interaction with Phe317 (Fig. 5 ▶ *b*).

## 4. Discussion   

A promising hit, compound **1a**, was identified from the H. Lundbeck compound collection by screening the displacement of ^3^H-NTS binding to sortilin in a scintillation proximity assay. We were initially suspicious of the activity of **1a** and were concerned that the hit could be spurious owing to assay interference resulting from some potentially problematical structural features of the molecule. A structural alert against the pan assay interference compounds (PAINS) filters for assay interference is triggered by molecules of type **1** owing to their potential to undergo a retro-Mannich reaction and liberate reactive species (Baell & Holloway, 2010 ▶). However, a sound structure–activity relationship was identified for six analogues of **1a** (**1b**–**1g**). Some variation in the length and size of the α-alkyl substituent of **1a** was allowed (**1b**–**1d**), whereas smaller (**1e** and **1f**) and larger (**1g**) substituents ablated activity (Fig. 1 ▶ *a*). The activity of **1a** was shown to be enantiospecific, with all activity residing in a single enantiomer, namely AF40431, whereas the corresponding antipode **1h** was inactive (Fig. 1 ▶ *c*). The direct binding of AF40431 to sortilin was confirmed by ITC. The observed *K*_d_ (0.70 µ*M*) is fivefold higher than the *K*_d_ of NTS (0.15 µ*M*), with the higher affinity of NTS originating from the hydrogen bond between Tyr11 of NTS and Lys227 of sortilin (Quistgaard *et al.*, 2009 ▶) and from contributions from the N-terminal region of NTS to binding (Quistgaard, 2014 ▶).

Crystals of the AF40431–sortilin complex were obtained by co-crystallization. A complete data set scaling to 2.7 Å resolution was collected and the structure was solved by molecular replacement. Despite belonging to the same space group as the sortilin–NTS complex structure, a different packing of the molecules (Supplementary Fig. S1), similar to that of the seleno­methionine-derivative and heavy-metal-derivative crystals of the sortilin–NTS complex, was observed (Quistgaard *et al.*, 2009 ▶). AF40431 is bound to sortilin at the NTS-binding site and the leucine moiety of the ligand is bound to sortilin in the same way as the leucine of NTS (Fig. 4 ▶).

Overall, the binding of AF40431 to sortilin is driven by four components: electrostatic interaction and hydrogen bonds between the carboxylic acid of AF40431 and Arg292, Ser283 and Tyr318, a hydrogen bond between the secondary amine of AF40431 and the carbonyl of Tyr318, hydrophobic inter­actions between the isopropyl group and Ser272, Phe281, Ile294 and Ile320, and π-stacking between the 2-pyrone and Phe317 (Fig. 5 ▶ *c*). The observed interactions are in good agreement with the free binding energy being driven by both enthalpic (electrostatic and hydrogen-bond interactions) and entropic (hydrophobic interaction) contributions (Fig. 2 ▶ *b*).

The selective stereochemistry of AF40431 is defined by the hydrogen bond between the secondary amine of AF40431 and the carbonyl of Tyr318, the electrostatic interaction with Arg292 and the hydrophobic interaction of the isopropyl group. The simultaneous interaction with these three components is excluded by the stereochemistry of **1h** (Fig. 5 ▶).

**Figure 6:**
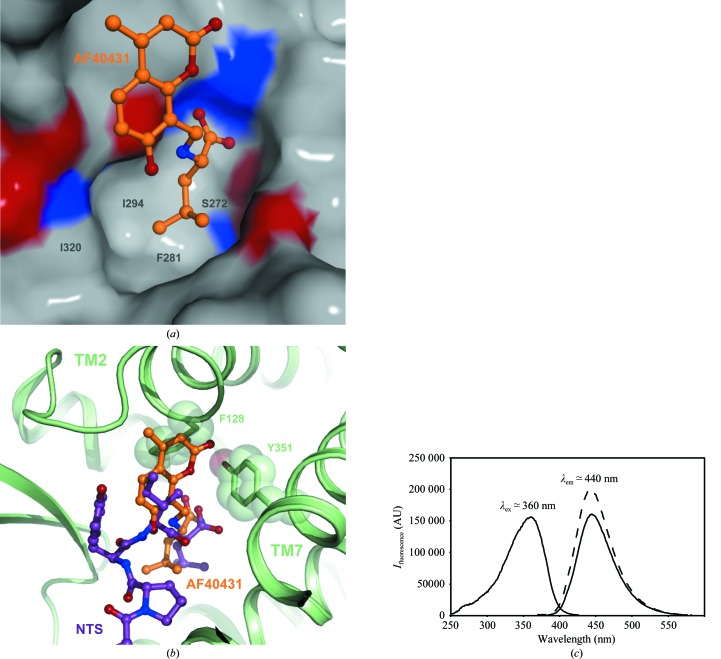
(*a*) The hydrophobic leucine-binding pocket. Sortilin is represented as a surface and is coloured according to atom type (C, N and O atoms in grey, blue and red, respectively). AF40431 is shown as a ball-and-stick representation in orange. The depth of the hydrophobic pocket is determined by Ser272, Phe281, Ile294 and Ile320. (*b*) Superposition of AF40431 and NTS in the structure of the NTSR1–NTS complex (PDB entry 4grv) represented as a cartoon in green (White *et al.*, 2012 ▶). The 4-methylumbelliferone moiety of AF40431 clashes with Phe128 in TM2 and Tyr351 in TM7. (*c*) Combined excitation and emission fluorescence spectrum for AF40431 (full lines) and the sortilin–AF40431 complex (dotted lines).

The structure–activity relationships obtained in the NTS scintillation proximity assay can be explained well by the size of the leucine-binding pocket of sortilin. The width of the pocket is determined by Ser272, Ile294 and Ile320, whereas the depth is determined by Phe281, which forms the ‘floor’ of the pocket (Fig. 6 ▶ *a*). The pocket allows substitutions of similar size, namely the isoleucine-type side chain of **1b** [CH(Me)Et] and the neopentyl and cyclohexyl substituents of **1c** and **1d**, respectively. The smaller isopropyl and methyl substituents of **1e** and **1f**, respectively, would not be expected to reach into the hydrophobic pocket and the bulkier benzyl substituent of **1g** (CH_2_Ph) would be expected to sterically clash with the ‘floor’ formed by Phe281 (Fig. 6 ▶ *b*).

The phenol group of the 4-methylumbelliferone moiety of AF40431 is located in the position corresponding to that of Ile12 of NTS in the sortilin–NTS complex (Figs. 5 ▶ *a* and 5 ▶ *b*) and, similar to Ile12, the phenol does not seem to contribute to the binding (Quistgaard *et al.*, 2009 ▶). A parallel π-stacking interaction is formed between the 2-pyrone of the 4-methyl­umbelliferone moiety of AF40431 and sortilin. An off-centre stacking is caused by the two electron-rich systems of 2-pyrone and Phe281 (Hunter & Sanders, 1990 ▶). The hydroxyl, ketone and methyl groups of 4-methylumbelliferone are not involved in interactions with sortilin and hence the structure–activity relationship should be explored for this two-ring system.

NTS binds to G-coupled neurotensin receptors (NTSRs) *via* amino acids 8–13, with the C-terminal leucine forming an electrostatic interaction with an arginine in the binding site (White *et al.*, 2012 ▶). Hence, off-target reactions of AF40431 with the NTSRs could be expected. However, the Ile12 pocket of NTSR1 is narrow and steric clashes between the 4-­methylumbelliferone moiety of AF40431 and Phe128 of transmembrane helix 2 (TM2) and Tyr351 of TM7 in NTSR1 were identified when AF40431 was superimposed on NTS in the NTSR1–NTS complex structure (White *et al.*, 2012 ▶; Fig. 6 ▶ *b*). No competition of NTS binding to NTSR1 was observed for AF40431 (IC_50_ > 50 µ*M*; data not shown).

The Calcein Blue moiety has previously been applied as a tracer in cellular and tissue studies (O’Malley *et al.*, 1999 ▶). The fluorescent property of Calcein Blue is retained in AF40431 and the fluorescence is not statically quenched by the π-­stacking with Phe317 upon binding to sortilin (Fig. 6 ▶ *c*). Hence, AF40431 could be applied as a molecular tracer of sortilin. The identification of AF40431 suggests that sortilin is ligandable by small molecules and thus provides a stimulus to further small-molecule ligand discovery efforts for sortilin.

## Supplementary Material

PDB reference: sortilin–AF40431 complex, 4msl

Supporting Information.. DOI: 10.1107/S1399004713030149/dw5079sup1.pdf

## Figures and Tables

**Table 1:** Data-collection and refinement statistics for the sortilin–AF40431 complex Values in parentheses are for the highest resolution shell.

Data collection	
Space group	*C*2
Unit-cell parameters (Å, °)	*a* = 160.9, *b* = 79.2, *c* = 111.7, β = 127.3
Resolution range (Å)	47.0–2.7 (2.85–2.70)
No. of unique reflections	30847
*R*_merge_ † (%)	4.5 (54.9)
*R*_p.i.m_ ‡ (%)	2.3 (27.4)
〈*I*/σ(*I*)〉	19.0 (2.3)
Completeness (%)	99.9 (99.7)
Multiplicity	3.9
Isotropic *B* factor (Wilson) (Å^2^)	84.4
Refinement	
Resolution range (Å)	47.0–2.7
No. of reflections	30842
*R*_work_/*R*_free_ § (%)	20.7/22.8
No. of atoms	
Protein (non-H)	5143
AF40431	23
Glycosylations	89
Water	67
*B* factors (Å^2^)	
Protein	97.7
AF40431	63.3
Glycosylations	106.6
Water	65.9
R.m.s. deviations	
Bond lengths (Å)	0.011
Bond angles (°)	1.260
Ramachandran plot, residues in (%)	
Most favoured region	97.8
Allowed region	2.2
Outlier region	0.0
PDB code	4msl

† *R*_merge_ = 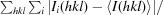 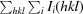, where *I_i_*(*hkl*) is the intensity of the *i*th observation and 〈*I*(*hkl*)〉 is the mean intensity of reflection *hkl*.

‡ *R*_p.i.m._ = 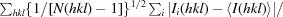 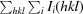, where *N*(*hkl*) is the multiplicity of reflection *hkl*.

§ *R* = 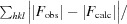 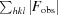, where *F*_obs_ and *F*_calc_ are the observed and calculated structure factors, respectively.
