# Supplementary material for: Identification of the first small-molecule ligand of the neuronal receptor sortilin and structure determination of the receptor–ligand complex
Source: Acta Crystallogr D Biol Crystallogr. 2014 Jan 29;70(Pt 2):451–60. doi: 10.1107/S1399004713030149 (PMC3940197; doi:10.1107/S1399004713030149)
Supplement: Supplementary file 1 [file d-70-00451-sup1.pdf]

SUPPORTING INFORMATION

Identification of the first small molecule ligand of the neuronal receptor sortilin; and structure determination of the receptor-ligand complex

Authors

Jacob Lauwring Andersen[a]*, Tenna Juul Schrøder[b], Søren Christensen[b], Dorthe Strandbygård[a], Lone Tjener Pallesen[c], Maria Marta García-Alai[a], Samsa Lindberg[b], Morten Langgård[b], Jørgen Calí Eskildsen[b], Laurent David[b], Lena Tagmose[b], Klaus Bæk Simonsen[b], Philip James Maltas[b], Lars Christian Biilmann Rønn[b], Inge E. M. de Jong[b], Ibrahim John Malik[b], Jan Egebjerg[b], Jens-Jacob Karlsson[b], Srinivas Uppalanchi[d], Durga Rao Sakumudi[d], Pradheep Eradi[d], Steven P. Watson[b]* and Søren Thirup[a]*

[a]The Lundbeck Foundation Research Centre MIND, Department of Molecular Biology and Genetics, Aarhus University, Gustav Wieds Vej 10C, Aarhus C, 8000, Denmark
[b]Neuroscience Drug Discovery, H. Lundbeck A/S, Ottiliavej 9, Valby, 2500, Denmark
[c]The Lundbeck Foundation Research Centre MIND, Department of Biomedicine, Aarhus University, Ole Worms Allé 3, Aarhus C, 8000, Denmark
[d]Medicinal Chemistry, GVK BioScience, Plot No. 28 A, IDA Nacharam, Hyderabad, 500076, India

Correspondence email: jla@mb.au.dk; stwa@Lundbeck.com; sth@mb.au.dk

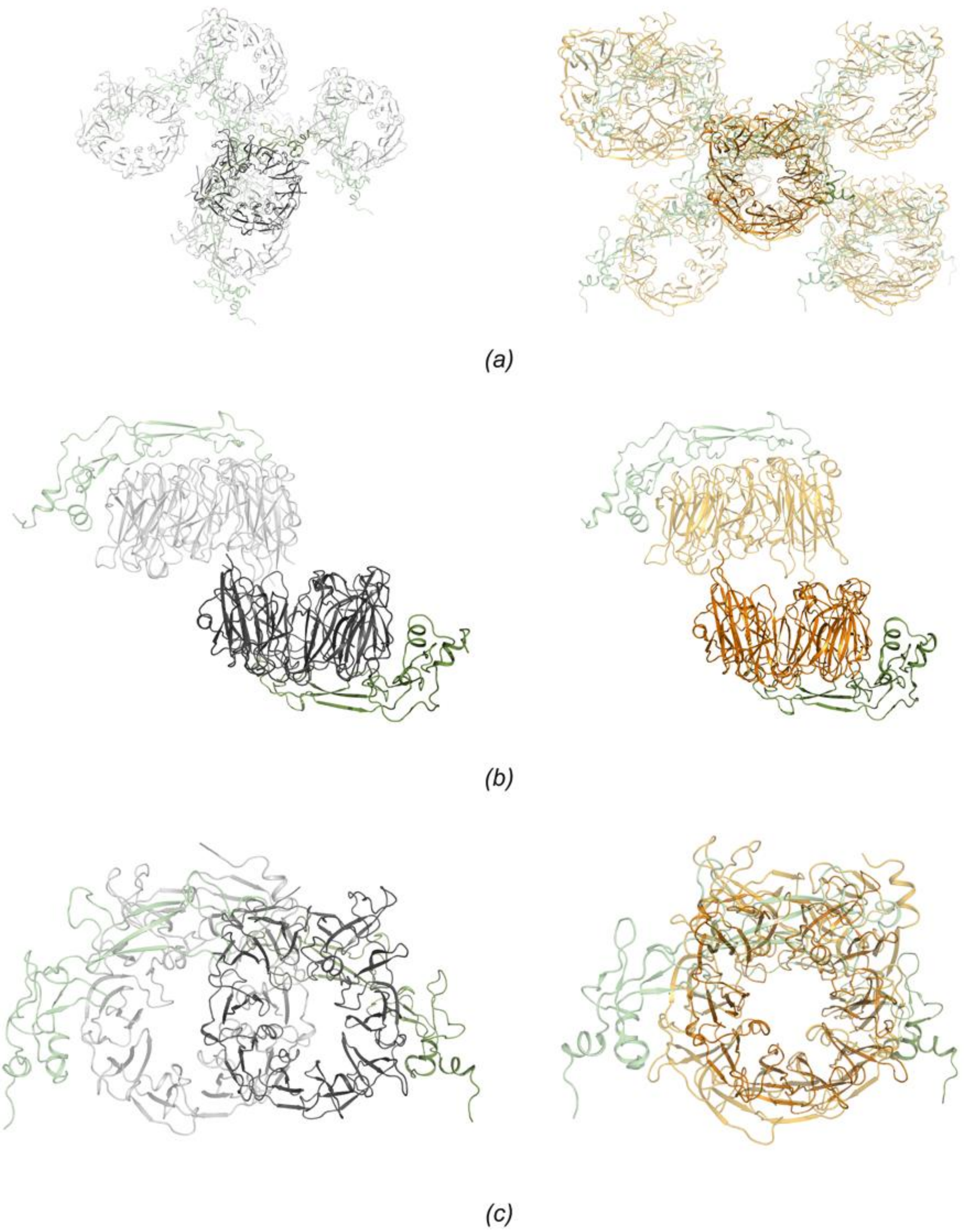

Figure S1: Overview of crystal packing. Differences in crystal packing between the sortilin-AF40431 complex (left figures with the β-propeller in grey and 10CC-domain in green) and the sortilin-neurotensin complex (right figures with the β-propeller in orange and 10CC-domain in green). **A**) Overview of the crystal packing with the symmetry related molecules (within a distance of 5 Å) in lighter colours. **B**) Side view displaying the off-centre packing in the sortilin-AF40431 complex crystal form. **C**) Top view displaying the off-centre packing in the sortilin-AF40431 complex crystal form.
